# Sex‐specific associations between AD genotype and the microbiome of human amyloid beta knock‐in (hAβ‐KI) mice

**DOI:** 10.1002/alz.13794

**Published:** 2024-04-04

**Authors:** Sage J. B. Dunham, Julio Avelar‐Barragan, Jason A. Rothman, Eric D. Adams, Gina Faraci, Stefania Forner, Shimako Kawauchi, Andrea J. Tenner, Kim N. Green, Frank M. LaFerla, Grant R. MacGregor, Mark Mapstone, Katrine L. Whiteson

**Affiliations:** ^1^ Department of Molecular Biology & Biochemistry University of California Irvine Irvine California USA; ^2^ Institute for Memory Impairments and Neurological Disorders (UCI MIND) University of California Irvine Irvine California USA; ^3^ Department of Anatomy and Neurobiology Developmental Biology Center University of California Irvine College of Medicine Irvine California USA; ^4^ Department of Molecular Biology & Biochemistry Department of Neurobiology and Behavior Department of Pathology and Laboratory Medicine School of Medicine Institute for Memory Impairments and Neurological Disorders (UCI MIND) University of California Irvine Irvine California USA; ^5^ Institute for Memory Impairments and Neurological Disorders (UCI MIND), Department of Neurobiology and Behavior School of Biological Sciences Center for Neural Circuit Mapping University of California Irvine Irvine California USA; ^6^ Institute for Memory Impairments and Neurological Disorders (UCI MIND) Department of Neurobiology and Behavior University of California Irvine Irvine California USA; ^7^ Department of Developmental and Cell Biology University of California Irvine Irvine California USA; ^8^ Department of Neurology University of California Irvine Irvine California USA

**Keywords:** 3xTg‐AD, Alzheimer's disease, cage effects, hAβ‐KI, late‐onset Alzheimer's disease, metagenomics, metabolomics, microbiome, mouse models for Alzheimer's disease, *Turicibacter*

## Abstract

**INTRODUCTION:**

Emerging evidence links changes in the gut microbiome to late‐onset Alzheimer's disease (LOAD), necessitating examination of AD mouse models with consideration of the microbiome.

**METHODS:**

We used shotgun metagenomics and untargeted metabolomics to study the human amyloid beta knock‐in (hAβ‐KI) murine model for LOAD compared to both wild‐type (WT) mice and a model for early‐onset AD (3xTg‐AD).

**RESULTS:**

Eighteen‐month female (but not male) hAβ‐KI microbiomes were distinct from WT microbiomes, with AD genotype accounting for 18% of the variance by permutational multivariate analysis of variance (PERMANOVA). Metabolomic diversity differences were observed in females, however no individual metabolites were differentially abundant. hAβ‐KI mice microbiomes were distinguishable from 3xTg‐AD animals (81% accuracy by random forest modeling), with separation primarily driven by *Romboutsia ilealis* and *Turicibacter* species. Microbiomes were highly cage specific, with cage assignment accounting for more than 40% of the PERMANOVA variance between the groups.

**DISCUSSION:**

These findings highlight a sex‐dependent variation in the microbiomes of hAβ‐KI mice and underscore the importance of considering the microbiome when designing studies that use murine models for AD.

**Highlights:**

- Microbial diversity and the abundance of several species differed in human amyloid beta knock‐in (hAβ‐KI) females but not males.
- Correlations to Alzheimer's disease (AD) genotype were stronger for the microbiome than the metabolome.
- Microbiomes from hAβ‐KI mice were distinct from 3xTg‐AD mice.
- Cage effects accounted for most of the variance in the microbiome and metabolome.

## 1 BACKGROUND

Like almost every niche on Earth, humans host complex communities of bacteria, fungi, viruses, archaea, and protozoa, forming the ecosystems collectively known as our microbiota.^1^, ^2^ Our microbial companions are critical to our daily health, but—when things go awry—they can cause or increase the severity of many diseases. Microorganisms in our gastrointestinal tract (which hosts > 1 trillion microbes^2^) help to extract nutrients from our food, modulate metabolism, promote immune function, and influence our overall mental health.^2^, ^3^ Gut microbes interact with the central nervous system through the microbiome–gut–brain axis, which is a bidirectional signaling network that operates using neural, endocrine, and immune links.^4^ Gut microbiome dysbiosis is now recognized as an indicator of pre‐clinical Alzheimer's disease (AD),^5^ and disruption of the gut microbiome via antibiotic treatment^6^ or fecal transplants^7^ can alter disease progression. Our understanding of the gut microbiome's influence on AD pathobiology is incomplete; however, a growing consensus is emerging around the role of the microbiome in mediating systemic inflammation, and, in turn, how inflammation influences AD.^8^

The relationship between gut microbiome composition and AD progression requires us to consider the microbial perspective when using murine models for AD research. Housing conditions, maternal links, diet, sex, and even the specific cage that the mice live in can affect microbiomes,^9^, ^10^, ^11^, ^12^, ^13^, ^14^, ^15^ which can in turn bias study results.^16^, ^17^ In their 2019 study of the relationship between gut microbiome dysbiosis, neuroinflammation, and AD progression in the transgenic 5xfAD mouse (a widely used model for early‐onset AD), Wang et al. found that convergent microbiomes among co‐housed wild type (WT) and 5xfAD animals led to changes in WT mouse immune cells, altered cytokine expression in the brain, and declines in discrimination learning that resembled the 5xfAD mice. The researchers were only able to make conclusions by housing the genotypes separately.^16^ Similarly, in their study of C1q or C5aR1 inhibition on the microbiome in the Arctic and Tg2576 mouse models of AD, Petrisko et al. found that co‐housing mice caused microbiome convergence, while separately housing the control and study arms allowed them to observe differences in the microbiome caused by AD genotype.^10^ Unfortunately, it is not as simple as housing genotypes separately, as keeping study arms in separate cages can cause cohort‐specific divergence in the microbiomes. In our recent examination of the longitudinal gut microbiome and metabolome of co‐housed 5xfAD mice, we found that between 50% and 80% of the variance in the microbiome arose from cage effects, while < 10% of the variance came from genotype.^9^ In a similar finding with the triply transgenic mouse model for early‐onset AD (3xTg‐AD), Borsom et al. reported that cage accounted for between 32% and 42% of the variance in the microbiome, while genotype accounted for an average of 8% of the variance.^18^ Cumulatively, this evidence points to the fact that researchers must, at a minimum, account for potential variations brought on by the microbiome. Failing to consider the microbiome can unknowingly bias the study results, especially when researching questions with links to the gut and systemic inflammation, such as late‐onset AD (LOAD).

Here we present our analysis of the longitudinal gut microbiome and metabolome of the human amyloid beta knock‐in (hAβ‐KI) mouse model for LOAD compared to age‐matched WT counterparts derived from the same genetic background (B6J). Developed in 2021, the hAβ‐KI mouse has no familial AD mutations and develops age‐dependent changes in behavior, inflammatory response, and synaptic plasticity that mimic the progression of LOAD seen in sporadic human cases.^19^ We compare the microbiomes of the 18‐month‐old animals to 3xTg‐AD mice of the same age. Our study aims to probe the relationship between the microbiome and metabolome of AD model animals and AD genotype, sex, and age. To our knowledge, this is the first microbiome‐focused study of a murine model for LOAD.

## 2 METHODS

Data collection and analysis was largely performed as described in our previous work.^9^ A brief description of the methods used, including any alterations, is given below.

### 2.1 Animal conditions and sample collection

Homozygous hAβ‐KI (hAbeta‐loxP‐KI knock‐in) mice have a modified amyloid precursor protein (*APP*) allele on chromosome 16, where three mutations were introduced into the mouse *APP* exon to encode the human Aβ^(1‐42)^ fragment. The modification also includes flanking the exon with loxP sites. As a result, these mice express the mouse APP protein with a “humanized” Aβ peptide sequence. They were housed together with same‐sex/genotype littermates after weaning (*n* = 41 mice in 13 cages). The hAβ‐KI control WT mice were derived from the same origin as the hAβ‐KI line (C57B6J). Cohorts were produced by breeding and in vitro fertilization (IVF).

The 3xTg‐AD mouse is a model of familial AD and expresses three homozygous mutations of *APP* Swedish, microtubule associated protein tau (*MAPT*) P301L (both transgenes are mapped on chromosome 2), and presenilin1 (*PSEN1*) M146V (on chromosome 12). After weaning, they were housed together with same‐sex/genotype littermates (36 mice in 16 cages). The 3xTgAD control WT mice were derived from the same origin as the 3xTgAD line (B6129). Cohorts were produced by breeding and IVF.

Animal experiments were conducted in compliance with all relevant ethical regulations for animal testing and research (as approved by the University of California Irvine [UCI] Institutional Animal Care and Use Committee). Animals were bred and aged in the Transgenic Mouse Facility at UCI and maintained in a 12/12‐hour light/dark cycle. All mice were fed LabDiet Irr6f (6% fat, 6% fiber) diet and sustained on pH 2.5–3.0 autoclaved water.

Immediately prior to sampling or sacrifice, the animals were isolated in individual cages for fecal collection. All working areas and tools were sterilized with 70% ethanol. Fecal pellets and cecum samples were deposited directly into 1.5 mL Eppendorf tubes. All sample tubes were placed on dry ice after collection and stored at −80°C until analysis.

**RESEARCH IN CONTEXT**

1. **Systematic review**: We reviewed available scientific literature relating to Alzheimer's disease (AD), murine models, and the microbiome using traditional online resources. Several recent publications describe the relationship between AD pathology and the microbiome, including those describing both mice and humans; however, no studies of murine models for late‐onset AD were found.
2. **Interpretation**: Our findings reveal genotype‐specific variations in female human amyloid beta knock‐in (hAβ‐KI) mice—but not males—and highlight the dramatic influence of co‐housing on the microbiome.
3. **Future directions**: hAβ‐KI mice are shown to be a promising platform for microbiome or diet‐specific interventional studies related to either diagnosing or mitigating AD. Our analyses highlight the need for careful, microbiome‐specific study design (including animal husbandry practices) and judicious consideration of the relationship between sex and the AD microbiome.

### 2.2 Sequence library preparation

All methods were performed as in our prior work.^9^ Briefly, DNA was extracted by Zymo Research Corp. using the ZymoBIOMICS‐96 MagBead DNA Kit (Zymo Research, Cat. # D4302) and quantified via the Quant‐iT PicoGreen dsDNA Assay Kit (ThermoFisher, Cat # P11496) read with a Synergy H1 Microplate reader (BioTek, Cat # BTH1M). Sequence libraries were prepared using the Nextera DNA Flex Library Prep Kit (Illumina, Cat. # 20018705) following a low volume variation of the standard protocol.^20^ Samples were prepared for polymerase chain reaction (PCR) with Kapa HiFi HotStart ReadyMix (Roche, Cat # 07958935001) and primers KAPA‐PCR‐F: 5′ – AATGATACGGCGACCACCG*A – 3′ and KAPA‐PCR‐R: 5′ – CAAGCAGAAGACGGCATACG*A – 3′. PCR was performed with an Eppendorf Mastercycler Nexus Gradient (Eppendorf, Cat # 2231000665) using standard thermal cycles for the Nextera Flex kit. The length distributions of the resulting sequence fragments were determined on an Agilent Bioanalyzer (Agilent, Cat # G2939BA). Sequence libraries were pooled based on DNA concentration and sequenced on an Illumina HiSeq4000 by Novogene Co., Ltd.

### 2.3 Metabolomics

Fecal and cecal material was sent on dry ice to the West Coast Metabolomics Center (WCMC) at University of California Davis for metabolomics analysis using the standardized WCMC extraction and analysis protocols for “Biogenic amines by HILIC‐QTOF MS/MS,” as previously described.^9^, ^21^ Approximately 4 mg of material was used from each sample, the final extract was dissolved in 100 μL acetonitrile/water (4:1, vol/vol) containing internal control standards, and 3 μL was analyzed on a SCIEX 6600 TTOF, in both positive and negative ionization. The resulting data was received from the WCMC as a table containing ion identifications and SERRF (Systematic Error Removal Using Random Forest) normalized peak areas.^22^ Only identified metabolites were used in this study.

Data for chemical taxonomy were generated using ClassyFire, an online chemical classification tool.^23^ The WCMC Chemical Translation Service (http://cts.fiehnlab.ucdavis.edu)^24^ was used to translate the chemical “identifiers” to an InChIKey list, which was uploaded to the ClassyFire website (http://classyfire.wishartlab.com) to generate classification into a list of 11 chemical superclasses.

### 2.4 Sequence data processing

As in our prior work,^9^ the sequence libraries were downloaded from the Novogene FTP website. The libraries consisted of 456 samples pooled into a single dual‐index paired‐end library and run across two lanes. The two paired‐end aggregate FASTQ files were parsed using BBDuk to trim adapters and remove artifacts, mouse DNA (GCA_000001635.8), and rat DNA (GCA_000001895.4). The cleaned files were demultiplexed using demuxbyname.sh inside of the BBmap suite.

Microbial taxonomy was assigned with Kraken v2.1.2.^25^ Additional parameters beyond the default included: “–gzip‐compressed,” “–minimum‐base‐quality 20,” “–use‐names,” and “–report‐zero‐counts”; Bracken v2.7 was used to estimate the number of reads originating from each species in each sample.^26^ Bracken parameters were set to “‐r 100” and “‐l S.” Individual reports generated by Bracken were merged using the included utility script, “combine_bracken_outputs.py,” to produce the final species count matrix.

### 2.5 Microbiome analysis

Processed sequence data were analyzed using RStudio version 1.4.1106^27^ and most graphics were created using the R package ggplot2.^28^ The heatmaps were generated from standard normalized abundance data using the pheatmap package^29^ with “average” clustering. Alpha diversity, non‐metric multidimensional scaling (NMDS), and permutational multivariate analysis of variance (PERMANOVA) were computed using the Vegan package.^30^ Significance of the alpha diversity metrics was assessed using the Kruskal–Wallis rank sum test with Benjamini–Hochberg false discovery rate (FDR) correction for eight measurements (one for each sex–cohort pair). Both NMDS and PERMANOVA were applied to the Bray–Curtis dissimilarity matrix. PERMANOVA was performed on all hAβ‐KI cohorts together by stratifying by cohort (age and sample type), and nesting Genotype and Sex within Housing_ID using the formula: adonis2(formula = data_subset ∼ (Genotype+Sex)/Housing_ID, data = meta_test, method = “bray,” permutations = perm, parallel = 32, by = “terms”). PERMANOVA was also performed on each individual sex‐specific hAβ‐KI cohort by nesting Genotype within Housing_ID and using the following formula: adonis2(formula = data_subset ∼ Genotype/Housing_ID, data = meta_test, method = “bray,” permutations = 999, parallel = 32, by = “terms”). PERMANOVA *P* values were Benjamini–Hochberg FDR corrected. Random forest was performed using the rfPermute package (v2.1.81)^31^ with a relative abundance threshold of 0.0001. Linear mixed‐effects (LME) model analysis was performed on relative abundance data using the lmer function in the lme4 package,^32^ a relative abundance threshold of 0.0001, Housing ID as the random effect, and a Benjamini–Hochberg FDR correction for repeated measurements.

### 2.6 Metabolomics analysis

Analysis of the metabolomics data was performed as described previously.^9^ Briefly, SERRF normalized data from the WCMC was uploaded into R and analyzed using a workflow like that used for the microbiome analysis. The Spearman correlations between the 100 most abundant metabolites and microbes were generated using the “cor” function in the stats package of R^33^ and the significance of the correlations were assessed by the Mantel test^30^ using the following formula on the Bray–Curtis distance matrices: mantel(mx.dist, otu.dist, method = “spearman”, permutations = 9999, na.rm = TRUE). Rows and columns are ordered by hierarchical clustering, and plotting was done using the pheatmap package.^29^

## 3 RESULTS

### 3.1 Overview of shotgun metagenomic sequencing and metabolomics

Our study included 41 animals from the hAβ‐KI line, with four samples for each animal: 4‐month fecal, 12‐month fecal, 18‐month fecal, and 18‐month cecal (164 samples total). Fecal and cecal samples were collected from 36 18‐month‐old animals from the 3xTg‐AD line. hAβ‐KI samples were subjected to both shotgun metagenomic sequencing and metabolomics (3 metagenomic and 19 metabolomic samples were omitted for inadequate material or poor resulting data quality), while 3xTg‐AD samples were subjected to metagenomics only. We obtained an average of 1.8 million (+/– 380,000) microbial reads per sample, 26% of which were successfully annotated. Approximately 99.8% of the annotated reads were bacterial, 0.03% were viral, and 0.1% were archaeal. For metabolomics, we observed a total of 4839 unique metabolic signatures, which included 458 named metabolites. Only named metabolites were considered in this analysis.

### 3.2 Most abundant microbes and metabolites

**FIGURE 1:**
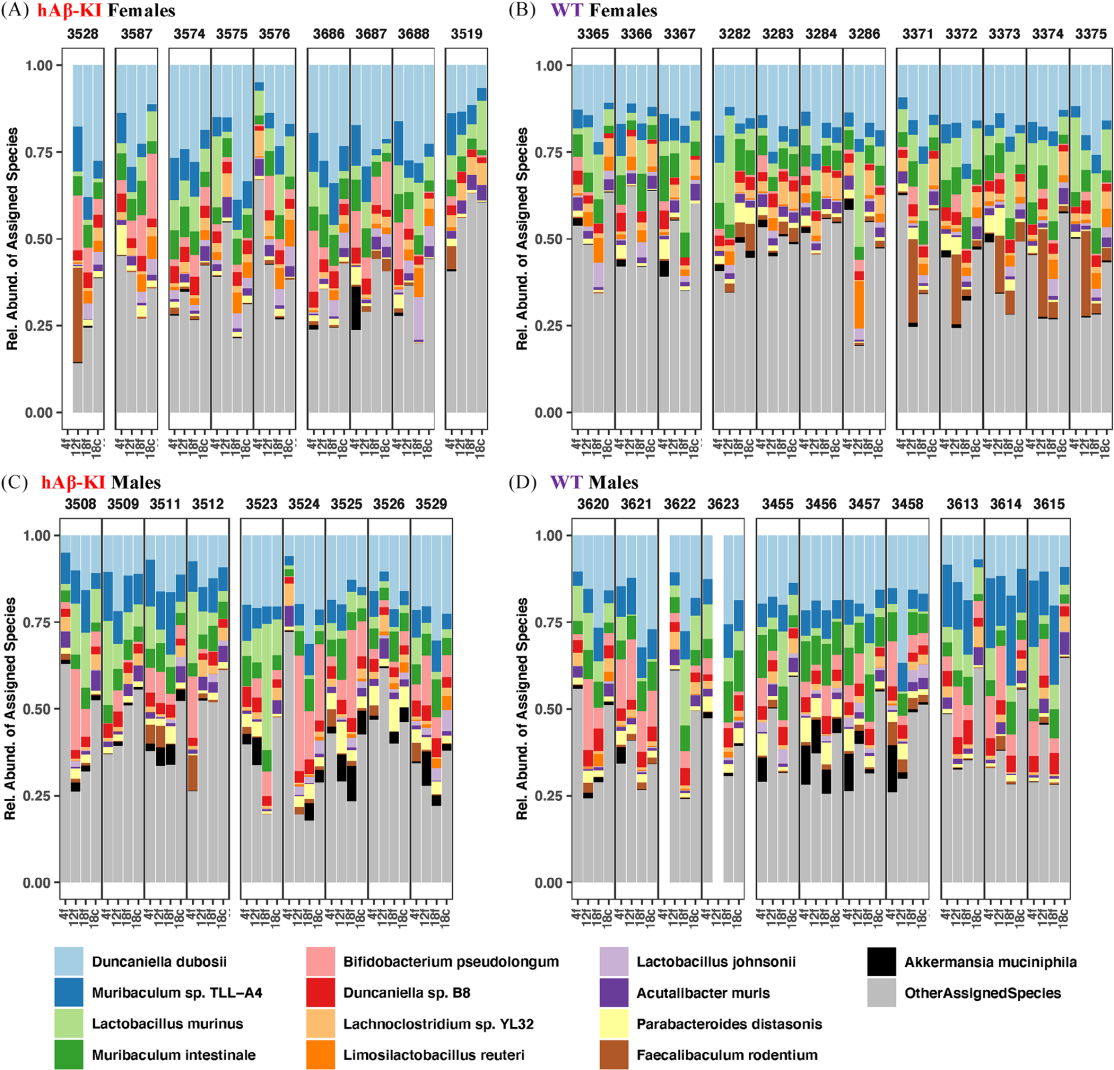
Most abundant microbes in (A) female hAβ‐KI, (B) female WT, (C) male hAβ‐KI, and (D) male WT mice. The samples are separated by mouse (denoted by the number above each black box; 9, 12, 9, and 11 mice for A, B, C, and D, respectively), cage of residence (clusters of boxes separated by whitespace, 5, 3, 2, and 3 cages for A, B, C, and D, respectively), and cohort (x‐axis labels, 4f = 4‐month fecal, 12f = 12‐month fecal, 18f = 18‐month fecal, and 18c = 18‐month cecal). Displayed are the 10 most abundant species in each grouping (13 total species) ordered by their mean abundances in the hAβ‐KI female mice. hAβ‐KI, human amyloid beta knock‐in; WT, wild type.

Figure 1 shows the relative abundances of the top 10 species in each genotype‐sex grouping across all hAβ‐KI microbiomes (i.e., hAβ‐KI females [Figure 1A], WT females [Figure 1B], hAβ‐KI males [Figure 1C], and WT males [Figure 1D]). Each bar represents the relative abundances of the top species in a single sample, and bars grouped within a black box represent all samples from a given mouse (4‐month fecal, 12‐month fecal, 18‐month fecal, and 18‐month cecal), and the whitespace‐separated mouse groupings show co‐housed mice.

There was a striking influence of co‐housing on microbiome composition, with microbial abundance and presence/absence dependent on the cage. For example, WT females in the final cage grouping (Figure 1B, mice IDs 3371–3375) all had a high abundance of *Faecalibaculum rodentium*, while this bacterium was largely absent from mice in other cages. Based on the trends shown in Figure 1B, *F. rodentium* was acquired by the mice in this cage somewhere between 4 and 12 months, and its relative abundance gradually declined thereafter. Another organism that showed the influence of co‐housing was *Akkermansia muciniphilia*, which had a high relative abundance in the 4‐month‐old WT males residing in the second cage (Figure 1D). Trends in genotype, sex, or consistent age‐associated differences are not readily apparent in this representation of the data. A similar analysis of the metabolomic data (Figure S1 in supporting information) showed that most of the named metabolites were organoheterocyclic compounds, organic acids (or derivatives), or lipids. Few discernible trends in genotype, sex, or age were observed.

Expanding our observational analysis to less abundant microbe and metabolite signatures revealed several trends, mostly pertaining to age and sample type (cecal vs. fecal). A heatmap of the 100 most abundant microbes (Figure S2A in supporting information) shows a general clustering between those microbes that were either more or less abundant in the 18‐month cecal samples relative to the fecal samples, with some clear sex‐specific effects (e.g., reduced *Alistipes* spp. in males relative to females). Several notable organisms are present on this plot, including two *Turicibacter* spp. (*T*. sp. H121 and *T. sanguinis*), which appeared at elevated abundances in 18‐month hAβ‐KI mice relative to WT animals. From a metabolomics perspective (Figure S2B) we see similar (but less pronounced) trends. A large group of metabolites near the bottom of the plot (tryptophan through glutamic acid) had higher abundance in 18‐month fecal and cecal samples relative to the younger mice, and another grouping near the top of the heatmap (octadecanoic acid through trimethylamine n‐oxide [TMAO]) were more abundant in the 4‐month samples. Broad metabolic trends relative to genotype and sex are difficult to observe in this representation of the data.

### 3.3 Correlation between microbes and metabolites

**FIGURE 2:**
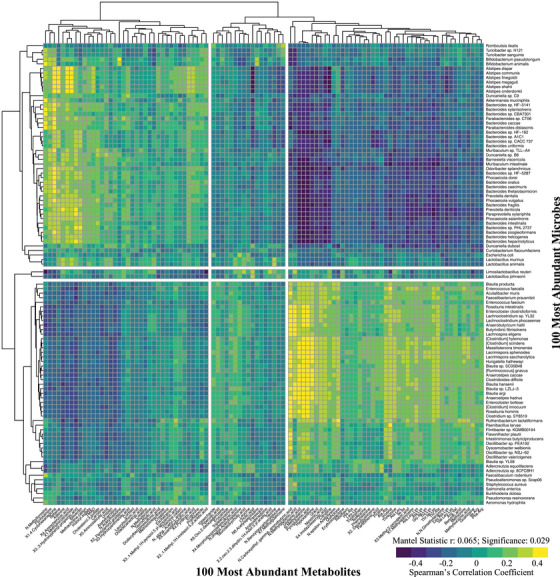
Spearman correlation between the 100 most abundant microbes (rows) and 100 most abundant metabolites (columns) in all longitudinal human amyloid beta knock‐in (hAβ‐KI) samples (161 samples from 41 animals). Each small square represents a correlation between the row and column variables, and is colored according to the correlation coefficient. Rows and columns are ordered by hierarchical clustering. The top 100 microbes and metabolites are significantly correlated (Mantel statistic: r = 0.065; significance = 0.029).

Spearman's correlations between the 100 most abundant metabolites and microbes at the species level showed several distinct clusters of co‐occurrence (Figure 2; Mantel statistic: r = 0.065, significance = 0.029). Segregating the heatmap into nine groups revealed two clusters of strong correlations (yellow clusters in upper left and lower right), two clusters of anticorrelations (blue region clusters upper right and lower left), and seven clusters in which the correlations were null or mixed. Several noteworthy observations can be drawn from these correlations. First, *Alistipes* and *Bacteroides* spp. showed similar correlation patterns and were positively correlated with ≈ 40% of the metabolites and negatively correlated with another 45%. The bottom cluster of microbes was more diverse and exhibited the opposite correlation pattern. Second, several smaller groupings of bacteria defied these trends. For example, *Limosilactobacillus reuteri* and *Lactobacillus johnsonii* (which formed their own distinct cluster near the center of the correlation heatmap) were positively correlated with the middle cluster of metabolites, and otherwise showed mixed correlation patterns with the remaining metabolite clusters. The five bacteria found at the top of the plot (*Romboutsia ilealis*, *Turicibacter* sp. H121, *Turicibacter sanguinis*, *Bifidobacterium pseudolongum*, and *Bifidobacterium animalis*) showed similar correlation patterns and were weakly correlated or weakly anticorrelated with most metabolites. Analogous correlation analyses applied to each sex, cohort, or genotype separately did not show statistically significant trends (Mantel significance > 0.05).

### 3.4 Alpha diversity of microbiomes and metabolomes

**FIGURE 3:**
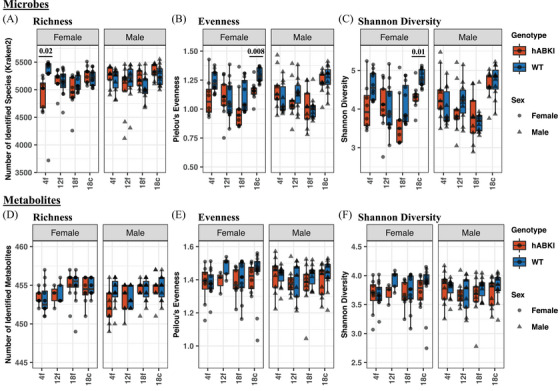
Alpha diversity of longitudinal (A‐C) microbiomes and (D‐F) metabolomes from hAβ‐KI and WT animals with males and females considered separately. Microbial evenness and Shannon diversity were reduced for hAβ‐KI mice relative to their WT counterparts in cecal samples from 18‐month females. Richness was also significantly reduced for 4‐month hAβ‐KI females relative to 4‐month WT females. x‐axis labels: 4f = 4‐month fecal, 12f = 12‐month fecal, 18f = 18‐month fecal, and 18c = 18‐month cecal. The microbiome and metabolome analysis included 161 and 145 samples, respectively, from 41 animals. *P‐values were computed using the Kruskal–Wallis rank sum test with Benjamini–Hochberg false discovery rate correction. hAβ‐KI, human amyloid beta knock‐in; WT, wild type.

The alpha diversities of the hAβ‐KI mice microbiomes and metabolomes were compared to their WT counterparts across all age groups, sample type, and sexes. As shown in Figure 3A, microbiome richness (the number of identified species) was similar for males and females across all age groups and genotypes, except for the 4‐month hAβ‐KI females, which had fewer detected species relative to age‐ and sex‐matched WT samples. Species evenness (Figure 3B) and Shannon diversity (Figure 3C; a diversity metric that includes both richness and evenness) were significantly lower in 18‐month hAβ‐KI females relative to their age‐ and sex‐matched WT counterparts. The difference was more pronounced in the 18‐month cecal microbiomes than the fecal microbiomes, and altogether absent from the males. Analogous diversity metrics applied to the metabolomes did not significantly vary with age, sex, or genotype (Figures 3D‐F).

### 3.5 Beta diversity of microbiomes and metabolomes

**FIGURE 4:**
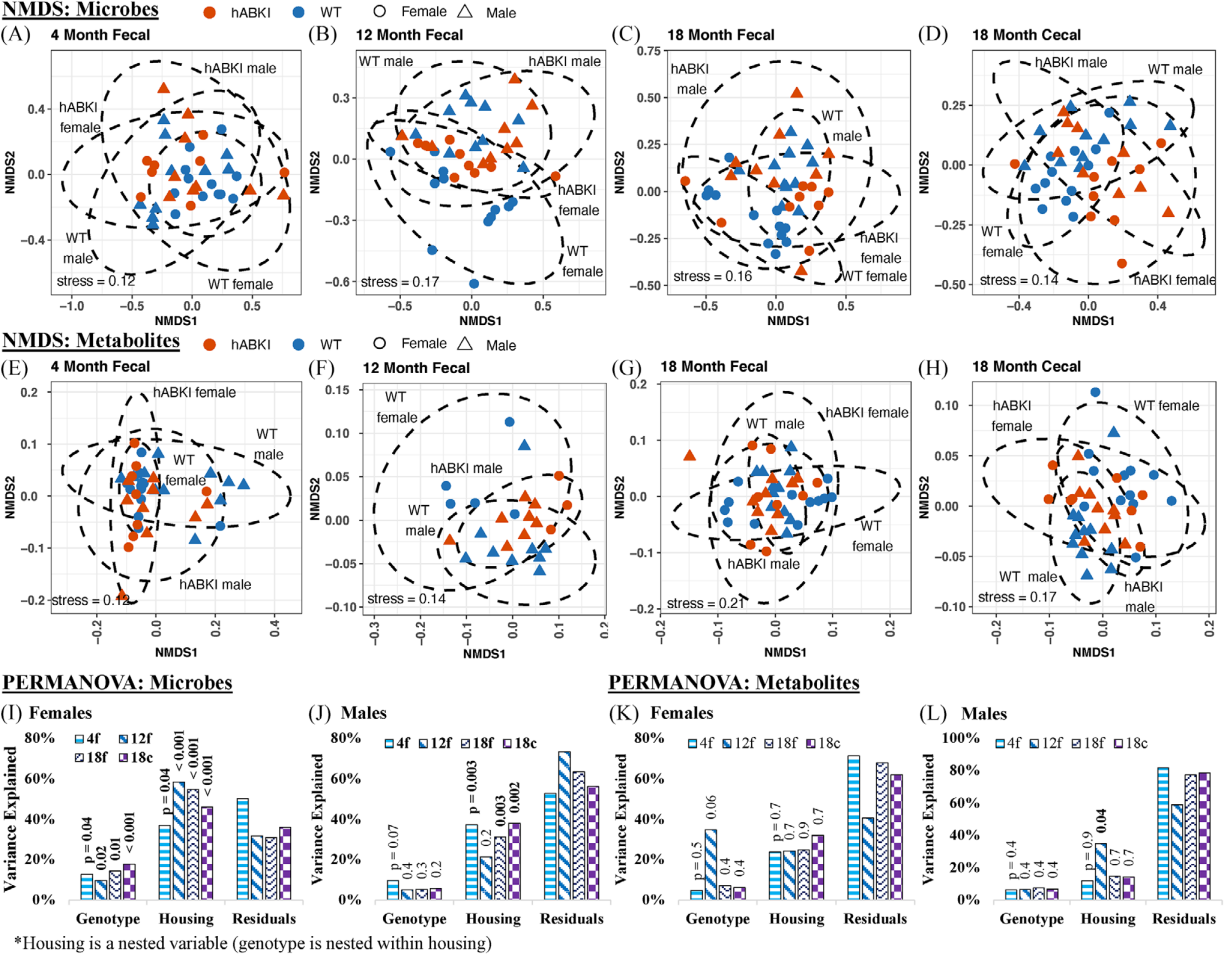
NMDS of Bray–Curtis dissimilarities of (A‐D) microbes and (E‐H) metabolites, and PERMANOVA of the microbes (I‐J) and metabolites (K‐L). Ellipses in (A)–(H) represent the 95% confidence interval for each genotype and sex grouping. The bars in (I)–(L) represent the magnitude of the PERMANOVA variance explained by each variable, with *P* values shown as a data label above each bar. The “Residuals” variable in (I)–(L) represents the variance unaccounted for by Genotype and housing. The microbiome and metabolome analysis included 161 and 145 samples, respectively, from 41 animals. PERMANOVA was performed on each sex‐specific hAβ‐KI cohort by nesting Genotype within Housing_ID and using the following formula: adonis2(formula = data_subset ∼ Genotype/Housing_ID, data = meta_test, method = “bray”, permutations = 999, parallel = 32, by = “terms”). The resulting PERMANOVA *P* values (text above the bars in I‐L) were adjusted with a Benjamini–Hochberg false discover rate corrected. hAβ‐KI, human amyloid beta knock‐in; PERMANOVA, permutational multivariate analysis of variance; WT, wild type.

NMDS of the Bray–Curtis dissimilarity matrices (a measure for beta diversity) was performed separately on the four cohorts (Figure 4A‐H), with 95% confidence interval ellipses drawn around each genotype–sex pairing (i.e., hAβ‐KI females, WT females, hAβ‐KI males, WT males). In both the microbiome (Figure 4A‐D), and metabolome (Figure 4E‐H) data, samples from different groupings overlapped, with slightly more separation for females than males.

PERMANOVA of the Bray–Curtis dissimilarity matrices was performed for both the microbiome (Figure 4I‐J) and metabolome data (Figure 4K‐L) with the males and females of each cohort treated separately. The female microbiome samples from each cohort (Figure 4I) varied significantly with respect to both genotype and housing ID, with genotype explaining 13%, 9.8%, 14%, and 18% of the variance in the 4‐month fecal, 12‐month fecal, 18‐month fecal, and 18‐month cecal samples. The finding with the female microbiomes stands in stark contrast to analogous analysis in males (Figure 4J), where genotype did not explain a significant portion of variation in any cohort (*P*adj > 0.05). Housing was a significant variable in all cohorts for both males and females (except for the 12‐month fecal males), where it accounted for as much as 58% of the variance in the microbiome.

For the metabolomes (Figures 4K‐L), housing accounted for a large proportion of the variance (> 10% in all cases) but was only significant for the 12‐month male fecal samples. Differences in the metabolome attributable to genotype were not significant.

### 3.6 Differentially abundant microbes and metabolites

Random forest modeling was performed on the microbes and metabolites found within the longitudinal hAβ‐KI samples for each cohort, with differentiation by each sex–genotype grouping (Figure S3 in supporting information). In the microbiome data (Figure S3A‐D), strong overlap and low predictive confidence were observed in all cases, with the possible exception of the 18‐month cecal samples (Figure S3D), which showed sex–genotype separation with 66% accuracy. Random forest modeling for the metabolomics data (Figure S3E‐H) showed a more pronounced separation for sex, but minimal separation by genotype for all cohorts.

**FIGURE 5:**
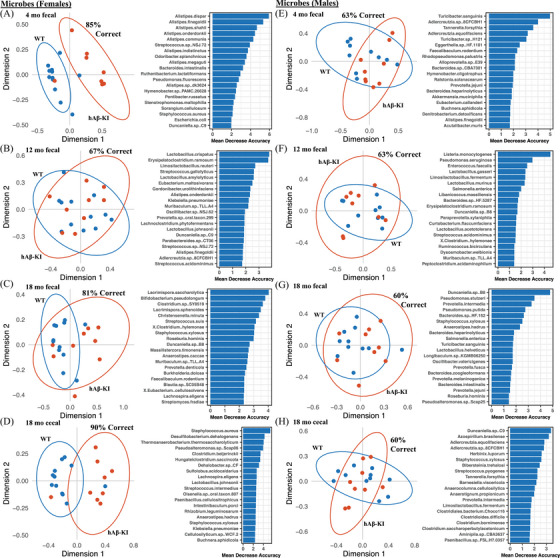
Random forest proximity plots and variables of importance for the longitudinal hAβ‐KI microbiome with females (A‐D) and males (E‐H) considered separately for each cohort. 95% confidence interval ellipses are drawn around each genotype grouping; 161 samples from 41 animals were included. hAβ‐KI, human amyloid beta knock‐in.

Because of the clear genotype differences observed elsewhere for females (and not males), we also performed random forest modeling on the females and males separately. As can be seen in the microbiome random forest analysis of Figure 5, three out of the four female cohorts exhibited clear separation by genotype (> 80% confidence), while all males did not (< 65% confidence). The clearest separation was observed for the 18‐month female cecal samples (90% confidence), with *Staphylococcus aureus, Desulfitobacterium dehalogenans, Thermoanaerobacterium thermosaccharolyticum*, and *Pseudoalteromonas* sp Scap06 identified as the variables of greatest importance.

Random forest modeling of the hAβ‐KI metabolomics data showed similar, although less pronounced, trends, with strong genotype differentiation in the females but not the males (Figure S4 in supporting information). For the females, the model predicted genotype with 80%, 88%, 70%, and 75% accuracy for the 4‐month fecal, 12‐month fecal, 18‐month fecal, and 18‐month cecal samples, respectively. For the males, the model achieved an accuracy of 50%, 76%, 55%, and 65% for the 4‐month fecal, 12‐month fecal, 18‐month fecal, and 18‐month cecal samples, respectively.

**FIGURE 6:**
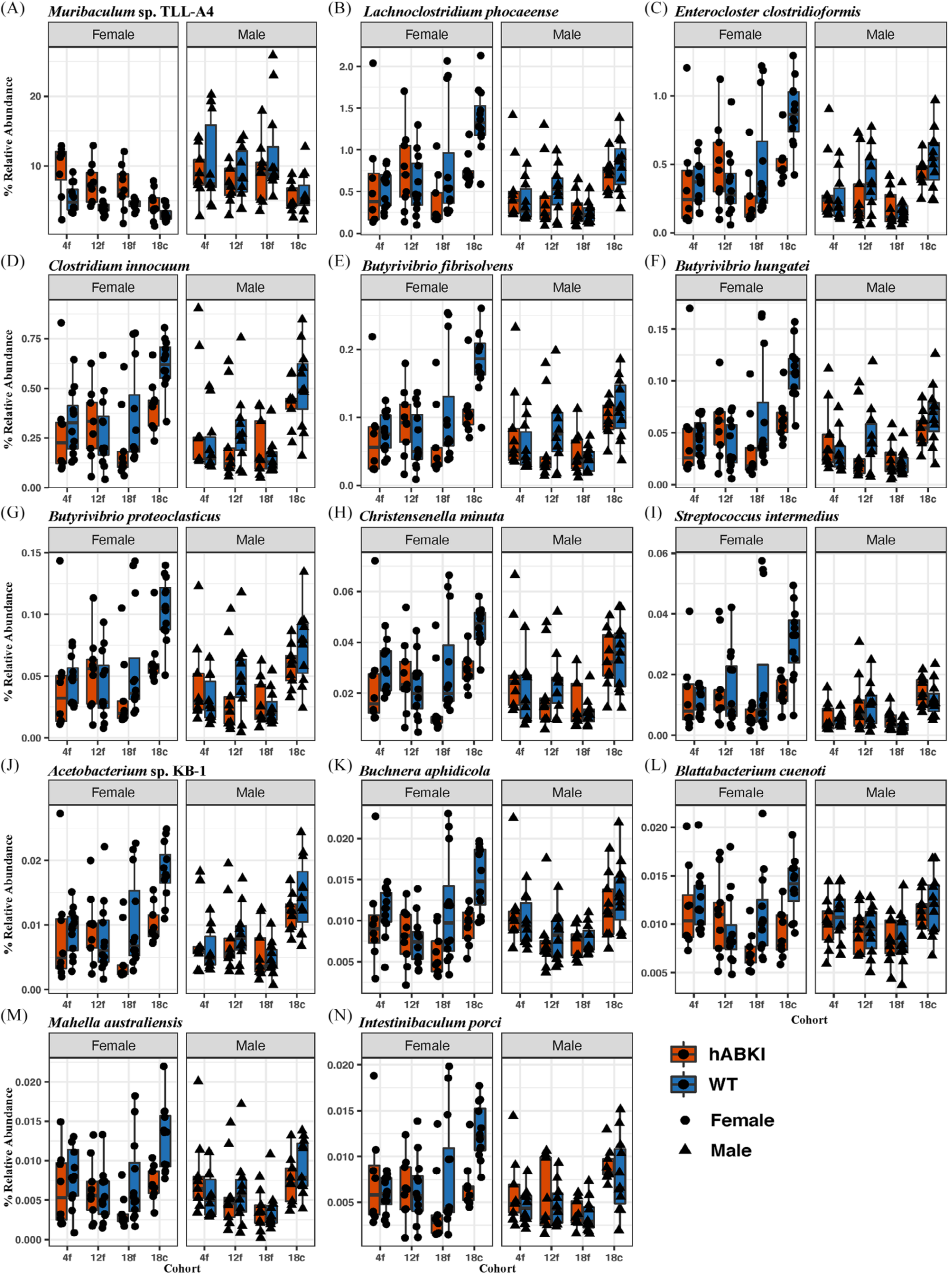
Microbes whose abundances significantly differ between genotypes as identified by LME with Benjamini–Hochberg false discovery rate correction; 161 samples from 41 animals were included. hAβ‐KI, human amyloid beta knock‐in; LME, linear mixed effects; WT, wild type.

We used a LME model to find microbes with significantly different abundances with respect to genotype when considering co‐housing as a compounding factor. We performed LME on all cohorts and sexes together, each sex (all cohorts), each cohort (both sexes), and each sex–cohort group. Differently abundant species (*P*adj < 0.05) were only observed for 18‐month female cecal samples (14 bacterial species) and 4‐month female fecal samples (*Muribaculum* sp. TLL‐A4 only). All differentially abundant species except for *Muribaculum* sp. TLL‐A4 exhibited a lower relative abundance in female hAβ‐KI mice relative to female WT mice (Figure 6). Remarkably, no differentially abundant species were found in male mice.

Performing the same set of LME analyses with respect to sex instead of genotype revealed only one bacterial species (*Limosilactobacillus reuteri*) to be differently abundant. *L. reuteri* was found at a significantly higher abundance in female mice relative to males (*P*adj = 3 × 10^−7^), a trend that held true for both hAβ‐KI and WT animals (Figure S5 in supporting information).

Applying LME modeling to the metabolomics data, no metabolites were significantly different between genotypes, while the abundances of seven metabolites were found to significantly differ between sexes after FDR correction: 2‐(1‐methyl‐1H‐imidazol‐5‐yl)acetic acid, benzoic acid, taurodeoxycholic acid, leucine, N‐methyl tyrosine, 4‐morpholinopropanesulfonic acid, and the di‐peptide Leu‐Lys.

Analyzing all cohorts together and using Mouse ID in place of Housing ID as the random effect in the LME model yielded a separate collection of differentially abundant species with respect to both genotype and sex (14 with respect to genotype when analyzing females only, and 39 with respect to sex). This method ignores the variability introduced by cage effects and therefore leaves ambiguity. For example, by this analysis, the abundance of *Bifidobacterium pseudolongum* was significantly lower in WT females relative to hAβ‐KI females (*P*adj = 0.016), but not consistently across all cages (Figure S6 in supporting information). Mice in two out of five cages showed *B. pseudolongum* abundances similar to the WT females (cages d & e in Figure S6). When performing LME with Housing ID as a random effect, spurious correlations between *B. pseudolongum* abundance and co‐housing were accounted for, and the model deemed the relationship to be above the significance threshold. Because of the ambiguity arising from cage effects, differential abundance analysis using Mouse ID as the random effect was not explored further.

### 3.7 Comparison of 18 mo hAβ‐KI and 3xTg‐AD microbiomes

We compared the microbiomes of the 18‐month hAβ‐KI mice to age‐matched animals from the 3xTg‐AD model for familial AD. The genetic background for each line was also included: B6J for hAβ‐KI (henceforth denoted as hAβ‐KI WT) and B6129 for 3xTg‐AD (henceforth denoted as 3xTg‐AD WT). The hAβ‐KI homozygous (HO) and hAβ‐KI WT samples used in this comparison were the same as those analyzed in prior sections of this article.

The two lines shared many of their most abundant microbial species, with more apparent taxonomic differences observed between sample types (i.e., fecal and cecal) than between genotypes or genetic background (Figure S7A‐D in supporting information). Many of the 3xTg‐AD WT samples (Figures S7C‐D) contained more *F. rodentium* than the other groupings, and generally had a higher proportion of the 12 most abundant species relative to the lower abundance species. These observations are also reflected in the alpha diversity metrics (Figure S7E‐G), which showed reduced richness and evenness for the 3xTg‐AD WT samples. Beta diversity analysis (NMDS of the Bray–Curtis dissimilarity matrix) showed large overlap for microbiomes from all genotypes and sexes, and strong separation by sample type (Figure S7H‐J). PERMANOVA showed that all variables examined were significant (*P* < 0.001) with variances of 2%, 2%, 5%, 15%, and 36% for genotype, line, sex, sample type, and cage (nesting variable).

**FIGURE 7:**
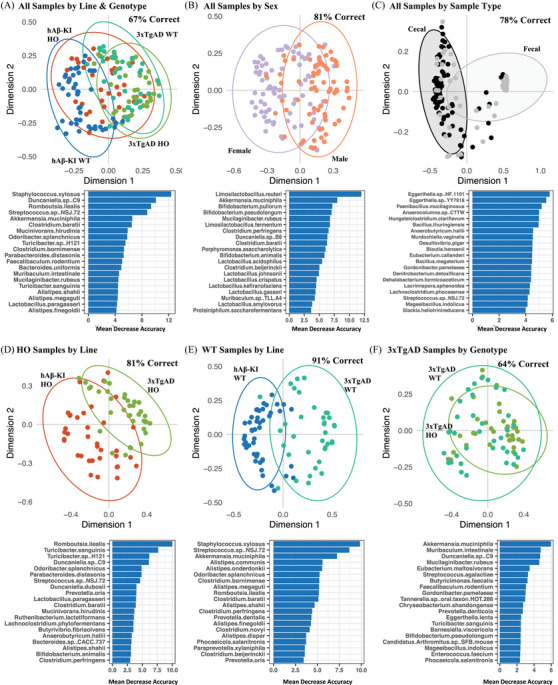
Random forest modeling of cecal and fecal microbiomes collected from 18‐month hAβ‐KI HO (*n* = 18), hAβ‐KI WT (B6J, *n* = 23), 3xTg‐AD HO (*n* = 18), and 3xTg‐AD WT (B6129, *n* = 18) mice. Modeling was performed with separation by line‐genotype grouping (A), sex (B), and sample type (C). Random forest was also used for the HO (D), WT (E), and 3xTg‐AD (F) samples separately. hAβ‐KI, human amyloid beta knock‐in; HO, homozygous; WT, wild type.

Random forest modeling showed separation by line and genotype (67% accurate, Figure 7A), sex (81% accurate, Figure 7B), and sample type (78% accurate, Figure 7C). A closer examination of the random forest model for line and genotype (Figure 7A) showed that most of the separation arose from genetic background (i.e., hAβ‐KI vs. 3xTg‐AD) rather than AD genotype (HO vs. WT). We explored this distinction further by focusing the model separately on the HO and WT samples from both genetic backgrounds (Figure 7D and 7E). Both the HO and WT samples showed distinct groupings, with 81% and 91% accuracy, respectively. Separation of the HO samples was driven by *Romboutsia ilealis*, *T. sanguinis*, *Turicibacter* sp. H121, and *Duncaniella* sp. C9 (mean decrease accuracy [MDE] > 5). Separation of the WT samples was largely driven by a different set of bacteria, namely *Staphylococcus xylosus*, *Streptococcus* sp. NSJ.72, *A. muciniphila*, *Alistipes communis*, *Alistipes onderdonkii*, and five other species with a MDE greater than 5. We also examined the relationship between the 3xTg‐AD HO and 3xTg‐AD WT samples (Figure 7F), which showed strong overlap between microbiomes from the two genotypes (64% accuracy).

The appearance of two *Turicibacter* spp. as top variables of importance in our random forest modeling (Figure 7D), and the prior studies highlighting a connection between *Turicibacter* spp. and AD^5^, ^9^, ^16^ led us to further investigate *Turicibacter* spp. abundance (Figure S8 in supporting information). The relative abundances of both *T. sanguinis* and *Turicibacter* sp. H121 were significantly greater in cecal and fecal material from hAβ‐KI HO mice relative to the 3xTg‐AD HO mice (by a Wilcoxon rank sum test, *P*adj < 0.05). The abundances of the two *Turicibacter* spp. were not significantly different comparing the HO and WT samples from within either genetic background (i.e., 3xTg‐AD HO vs. 3xTg‐AD WT, or hAβ‐KI HO vs. hAβ‐KI WT), or comparing the hAβ‐KI WT mice to the 3xTg‐AD WT mice.

## 4 DISCUSSION

We examined the composition of the longitudinal microbiome and metabolome in the hAβ‐KI mouse model for LOAD, with consideration of sex, age, and cage effects.

### 4.1 Female hAβ‐KI mice harbor distinct microbiomes, while male hAβ‐KI mice do not

AD‐specific differences are observed in female hAβ‐KI microbiomes but not in males. These sex‐specific trends are present in our alpha and beta diversity metrics (Figures 3 and 4), our random forest analysis (Figure 5), and the LME modeling for differentially abundant species (Figure 6). The PERMANOVA results (Figure 4I‐L) are particularly illustrative of this dichotomy, with genotype‐specific differences in beta diversity accounting for as much as 18% of the variance in the female microbiomes.

This finding partially recapitulates the sex‐specific results of other studies of the AD microbiome. For example, in their 2019 study of calorie restriction in Tg2576 mice, Cox et al. found more substantial age‐related microbiome changes in female mice, which were directly linked to differential Aβ levels.^34^ Some studies have chosen to use only female mice, presumably to increase the effect size.^18^ As a cautionary point to excluding males, a recent preprint studying the microbiota–microglia–amyloid axis only observed effects in male mice.^35^ Sex‐specific differences in people with AD are also well known, with females disproportionately diagnosed with AD.^36^ As of this writing, sexual dimorphism in hAβ‐KI mouse cognition, behavior, or AD severity has not been otherwise observed.

### 4.2 Female hAβ‐KI microbiomes diverge as the mice age, particularly in the cecum

The differences in female hAβ‐KI microbiomes with respect to genotype generally increase with age and are most pronounced in the cecal samples. This trend of an age‐dependent increase is expected for a mouse model that recapitulates a progressive disease. hAβ‐KI mice exhibit progressively worsening disease pathology, including age‐dependent impairments in cognition, brain volume, and inflammation.^19^

### 4.3 Female hAβ‐KI gut metabolomes are more similar than microbiomes

Our results suggest that the female hAβ‐KI fecal microbiomes vary more between genotypes than do the metabolomes. Remarkably, even the 4‐month hAβ‐KI females exhibited significantly different microbiome beta diversity compared to their WT counterparts (PERMANOVA *P*adj = 0.04, *R* ^2^ = 13%). This suggests that gut microbiome‐based diagnostic tools may be more promising than those based on the gut metabolome. Plasma metabolome‐based diagnostic tools for pre‐clinical AD are under development,^37^, ^38^ and other studies have indicated the potential use of gut microbiome biomarkers for diagnostics.^39^, ^40^

### 4.4 Differentially abundant microbes are found only in females

LME modeling identified 14 microbes (all anaerobes) whose abundances differed between genotypes (Figure 6). Aside from *Muribaculum* sp. TLL‐A4 (which was elevated in hAβ‐KI females), all microbes were present at significantly lower abundances in 18‐month hAβ‐KI females relative to age‐ and sex‐matched controls. No species were found to be differentially abundant in males. All microbes except for *Muribaculum* sp. TLL‐A4 were found at low abundances, with several (7/14) having relative abundances < 0.1%. These rare species may be an indicator of underlying disease physiology and may present diagnostic value.

### 4.5 Microbiomes of late and early onset AD models are distinguishable by PERMANOVA and random forest

Microbiomes from the early and late onset AD models (3xTg‐AD and hAβ‐KI) showed similar alpha diversity but were distinguishable by PERMANOVA and random forest. Genotype and line each accounted for ≈ 2% of the PERMANOVA variance, a robust finding in the context of both host‐associated and environmental microbiome studies, in which 2% variance is comparable to other factors important for microbiome composition such as age or antibiotic use.^41^, ^42^ Random forest (Figure 7) distinguished the two lines with accuracies of 81% and 91% for hAβ‐KI HO versus 3xTg‐AD HO and hAβ‐KI WT versus 3xTg‐AD WT, respectively, further suggesting that genetic background drives microbiome composition. Separation of the HO samples was driven by many of the same microbes (*Romboutsia*, *Turicibacter*, *Turicibacter*, and *Duncaniella*) that were found to be differentially abundant in the 5xfAD mouse.^9^ Perhaps surprisingly, our analysis did not reveal genotype‐specific differences between 18‐month 3xTg‐AD animals. As the 3xTg‐AD mouse was not the primary focus of our study, we leave further exploration of this finding to others and refer the reader to Borsom et al.^18^ Direct comparison of hAβ‐KI and 3xTg‐AD to the 5xfAD mice from our prior study^9^ was hindered by batch‐to‐batch variation in sequencing runs.

### 4.6 *Turicibacter* spp. and AD

*Turicibacter* spp. were more abundant in the cecal and fecal material of hAβ‐KI HO animals relative to those from the early onset AD model (3xTg‐AD HO) (Figure S8). The differences in *Turicibacter* spp. abundance within each line were not significant. *Turicibacter* has previously been associated with AD, both in 5xfAD mice (where *Turicibacter* spp. are depleted in older mice with AD pathology)^9^ and in humans, where it is depleted in people with AD relative to healthy counterparts.^5^ *Turicibacter* spp. are established mediators of the gut–brain axis in AD, through both the consumption of serotonin and the regulation of its production.^43^, ^44^ Although *Turicibacter* spp. are at relatively low abundances (<1%), the consistent emergence of these microbes as top variables of importance suggests their importance in AD.

### 4.7 Some rare taxa may have dietary or spurious database‐related origins

Taxonomic assignments for several species (including some found to be differentially abundant) may be spurious. We used a metagenomic sequencing pipeline (Kraken) that provides thousands of species, and not all assignments may be accurate. For example, *Blattabacterium cuenoti* and *Buchnera aphidicola* are obligate endosymbionts of insects and therefore unlikely to exist outside of their hosts.^45^, ^46^ DNA from these organisms may have come from the mouse chow, but their differential abundance is still unexplained. Although it has been reported as a low abundance species in human fecal microbiomes,^47^ another dubious assignment is *Mahella australiensis*, which is a thermophile originally isolated from an Australian oil well.^48^

### 4.8 Most of our metagenomic reads and metabolomic ions remain unassigned

Only 26% of our shotgun microbial reads matched sequences in the Kraken database, and only 10% of the metabolites detected with mass spectroscopy could be identified. The large proportion of unknowns is typical for omics methods, even in a well‐studied environments such as a mouse gut.^49^, ^50^ Further analysis of these unidentified signals may unearth important biomarkers, or even causative agents, for AD.

### 4.9 Cage effects obscure potentially important trends

A universal finding of microbiome research (including in humans) is that most microbiome variance is dictated by the subject and the housing unit in which the subject resides.^51^, ^52^, ^53^ These effects are even larger in coprophagic animals, who develop similar microbiomes to their cage‐mates. Small, unintended variations in the microbiome can alter seemingly unrelated study results, and therefore the researcher may be unable to determine whether a result is the true effect of the test parameter or a manifestation of the cage effect. Microbiome‐informed animal husbandry practices, clever experimental design, and creative statistical modeling are needed to tease out accurate results.^11^, ^14^, ^17^, ^54^, ^55^, ^56^

We found here that cage effects accounted for a plurality of the variance in the microbiome (37%–58% by PERMANOVA); however, we identified differentially abundant species by including housing ID as a random effect in our LME model. Cage effects were also substantial for the metabolome (21%–58%) but they were not significant (*P* > 0.05 by PERMANOVA). High variance explained with a lack of significance can occur when the PERMANOVA model does not fit the data well, or when there is inadequate variance in the replicates. Cage effects likely obscure relevant associations between the microbiome/metabolome composition and AD. For example, *Bifidobacterium pseudolongum* (Figure S6) appears to be differentially abundant at first glance but is ultimately an ambiguous result when cage effects are considered.

Kim et al. provided several suggestions on how best to design microbiome‐focused experiments, including maximizing the number of cages and treating the cage as a variable in any statistical analysis.^56^ More cages may increase expense, which will likely constrain the number of animals. Others have obtained favorable results through randomized cohousing, which can enable statistical disentanglement of the effects of genotype and housing.^17^ Cage effects may also be mitigated through intentional microbiome design. Inoculation with “wilding” microbiomes increases resilience,^57^ potentially preventing the unintentional microbiome‐induced perturbation of mouse physiology, behavior, and study outcomes. Wildling microbiomes may also better represent human immune response.^58^

## 5 CONCLUSIONS

We examined the longitudinal microbiome and metabolome of a mouse model for LOAD (hAβ‐KI) and explored the association with AD genotype, age, and sex. AD‐dependent differences were observed in gut microbial diversity and taxonomic composition, but only in 18‐month females, suggesting that the impact of hAβ‐KI genotype on the gut microbiome is more pronounced in females than males. Unsurprisingly, we also found that a plurality of the microbiome and metabolome variance is attributable to cage affects. Animals that are housed together share similar microbiomes, regardless of other factors. Considering housing design and early microbial exposures in LOAD animal model studies is imperative for our continued development of robust animal models of AD.

## CONFLICT OF INTEREST STATEMENT

The authors report no conflicts of interest. Author disclosures are available in the Supporting Information.

## CONSENT STATEMENT

No human subjects were used in this work and consent was not necessary.

## Supporting information

Supporting Information

Supporting Information
