# Supplementary material for: Sex‐specific associations between AD genotype and the microbiome of human amyloid beta knock‐in (hAβ‐KI) mice
Source: Alzheimers Dement. 2024 Apr 4;20(7):4935–50. doi: 10.1002/alz.13794 (PMC11247698; doi:10.1002/alz.13794)
Supplement: Supplementary file 1 — Supporting Information [file ALZ-20-4935-s002.pdf]

Sex-specific associations between AD genotype and the microbiome of human amyloid beta knock-in (hAβ-KI) mice

Sage J. B. Dunham[a*], Julio Avelar-Barragan[a], Jason A. Rothman[a], Eric D. Adams[a], Gina Faraci[a], Stefania Forner[b], Shimako Kawauchi[c], Andrea J. Tenner[d], Kim N. Green[e], Frank M. LaFerla[f], Grant R. MacGregor[g], Mark Mapstone[h], & Katrine L. Whiteson[a*]

*Address correspondence to Sage Dunham (sjdunham@uci.edu) and Katrine Whiteson (katrine@uci.edu).

Contents:

Figure S1: Metabolic superclasses
Figure S2: 100 most abundant microbes and metabolites
Figure S3: Random forest for microbiome and metabolome
Figure S4: Random forest for metabolomes with females and males considered separately
Figure S5. Abundance of *Limosilactobacillus reuteri*
Figure S6. Relative abundance of *Bifidobacterium pseudolongum* in female mice
Figure S7. Species-level taxonomy and diversity of 18 month hAβ-KI and 3xTg-AD microbiomes segregated by sex and sample type (cecal and fecal)
Figure S8. Analysis of *Turicibacter* spp. relative abundance

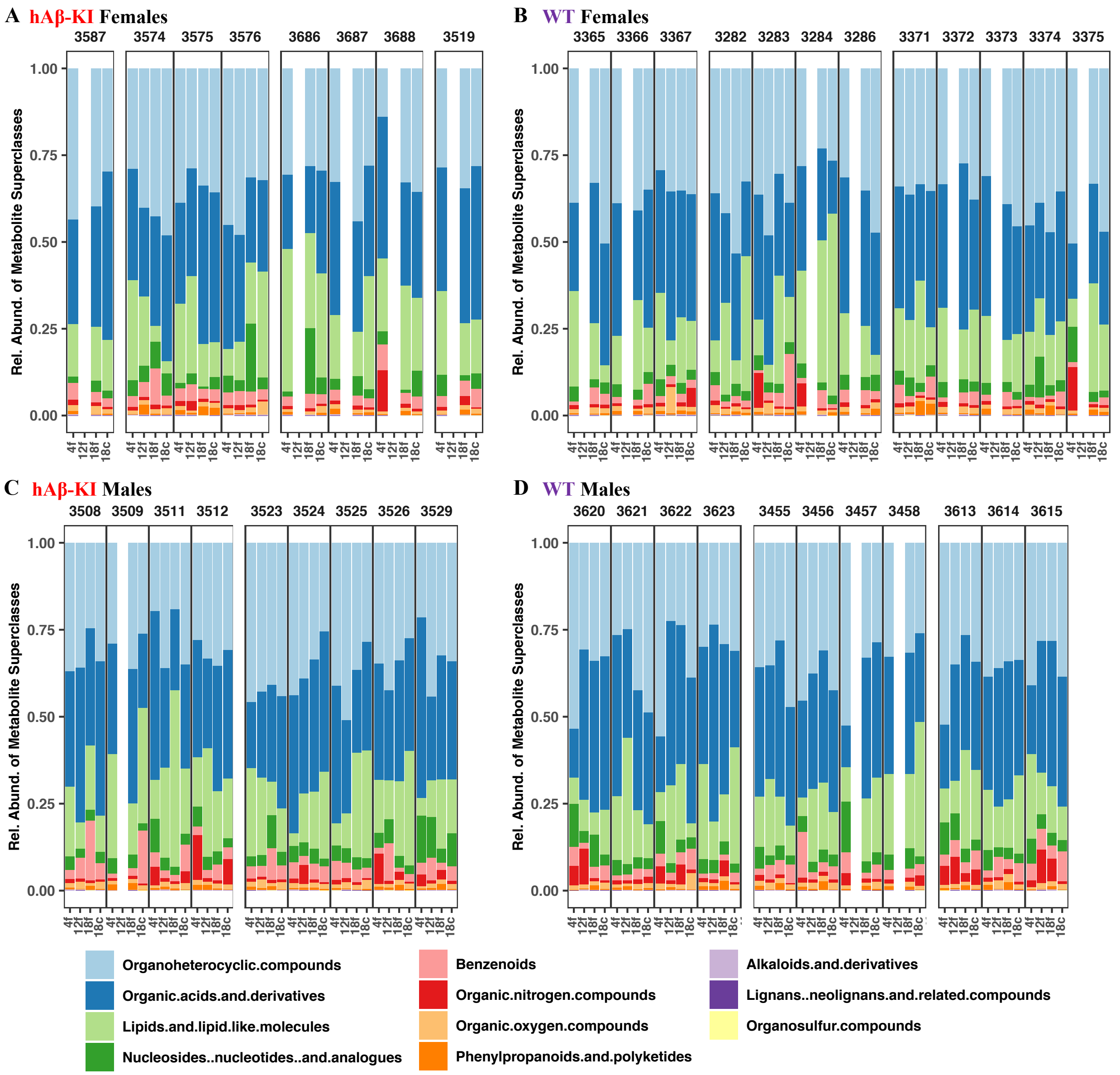

Figure S1: Metabolic superclasses in (**A**) female hAβ-KI, (**B**) female WT, (**C**) male hAβ-KI, and (**D**) male WT mice. The samples are separated by mouse (denoted by the number above each black box), cage of residence (clusters of boxes separated by whitespace), and cohort (x-axis labels, 4f = 4 month fecal, 12f = 12 month fecal, 18f = 18 month fecal, and 18c = 18 month cecal). Displayed are the ten metabolic superclasses ordered by their mean abundances in the hAβ-KI female mice.

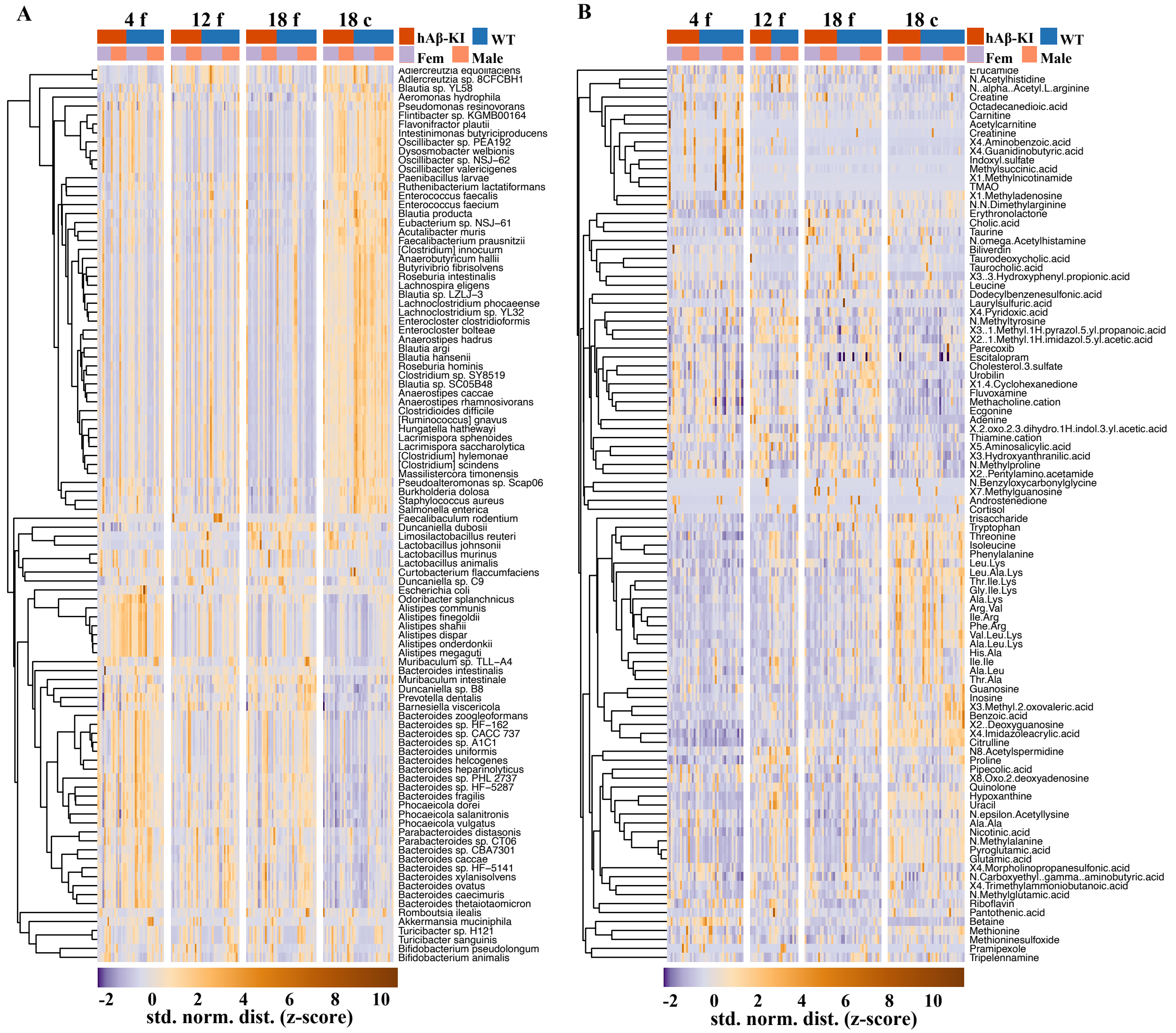

Figure S2: 100 most abundant (**A**) microbes and (**B**) metabolites. The samples (columns) are sorted by cohort, genotype, and sex, and the microbes and metabolites (rows) are allowed to cluster based upon abundance patterns. The color scale represents abundance (standard normal distribution). The hierarchical clustering of microbes and metabolites is represented by the dendrograms to the right of each figure.

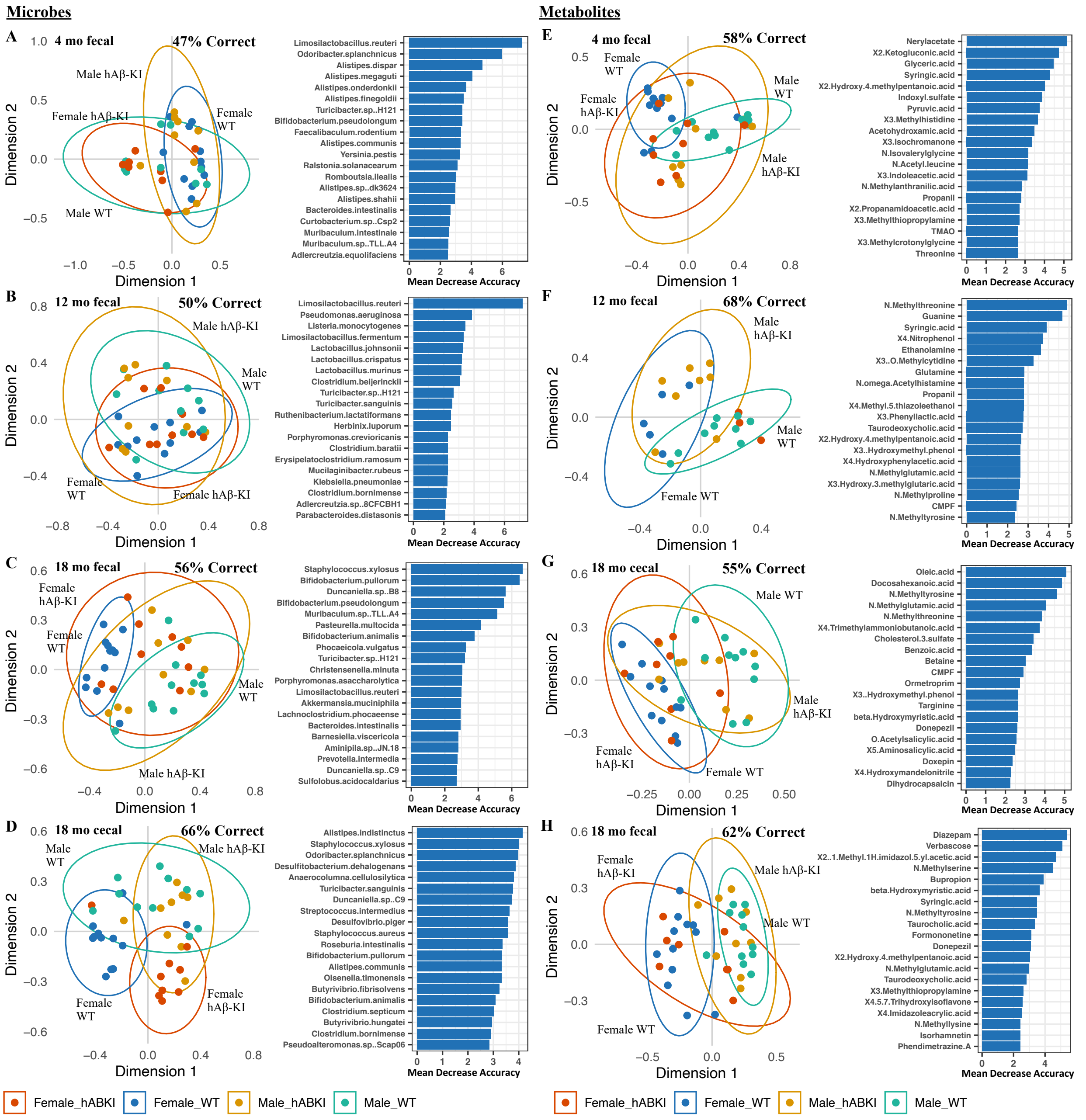

Figure S3: Random Forest proximity plots and variables of importance for the longitudinal hAβ-KI microbiome (**A-D**) and metabolome (**E-H**) with separate analysis for each cohort. 95% confidence interval ellipses are drawn around each sex-genotype grouping, with the exception of the female 12 month hAβ-KI samples, which had too few data points to draw an ellipse.

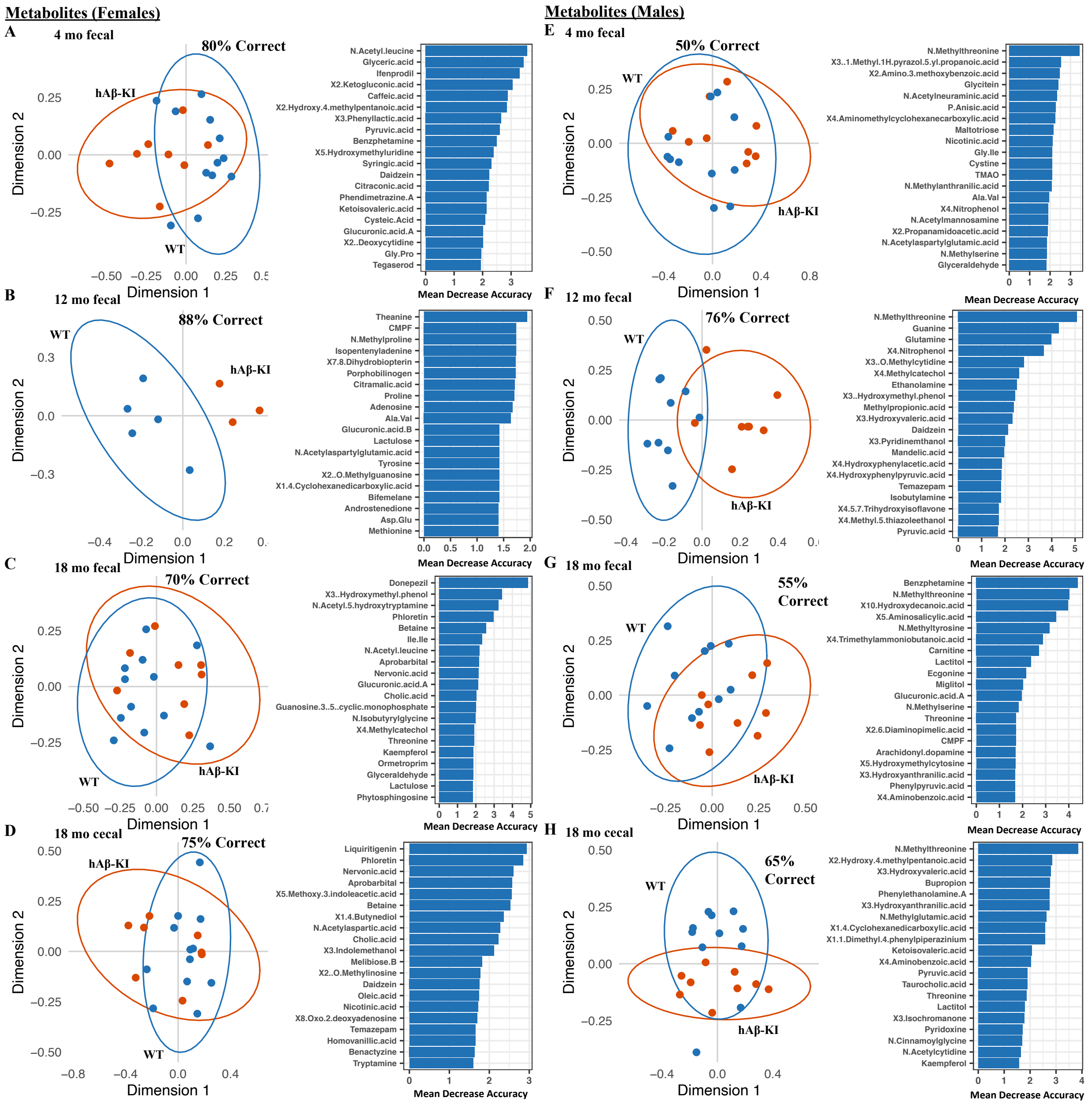

Figure S4: Random Forest proximity plots and variables of importance for hAβ-KI metabolomes with females (**A-D**) and males (**E-H**) considered separately for each cohort. 95% confidence interval ellipses are drawn around each genotype grouping except for 12 month females, which had too few samples to enable ellipses.

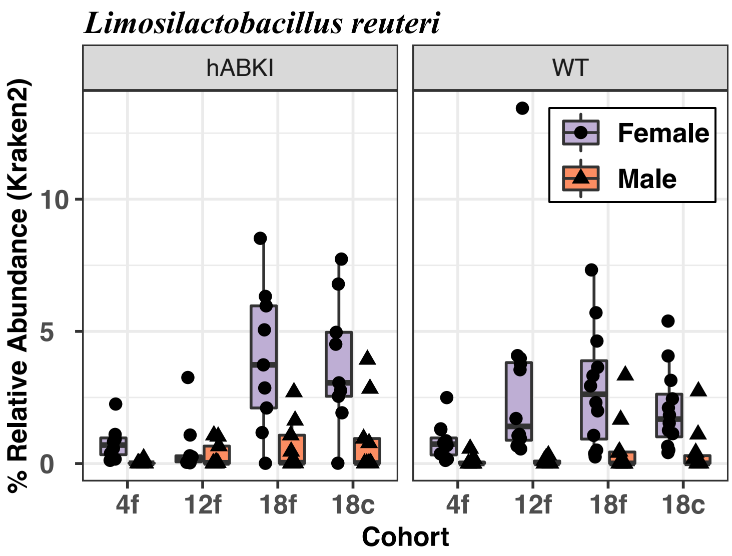

Figure S5. *Limosilactobacillus reuteri*, the only microbe whose abundance significantly differed between sexes by LME.

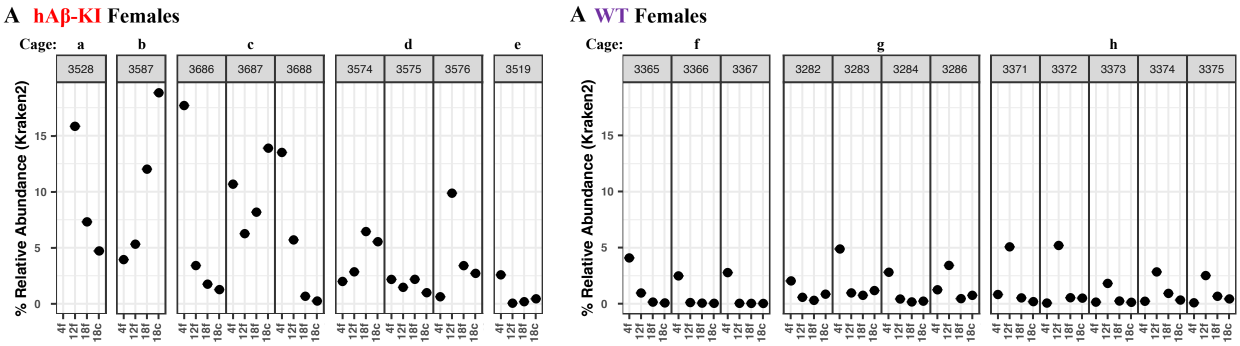

Figure S6. Relative abundance of *Bifidobacterium pseudolongum* in female mice. All samples (4f, 12f, 18f, and 18c) from each mouse (numbers in grey boxes on top of plot) are shown in separate black boxes with whitespace separating co-housed mice. Mice in cages a, b and c exhibited a higher abundance of *B. pseudolongum* relative to other cages.

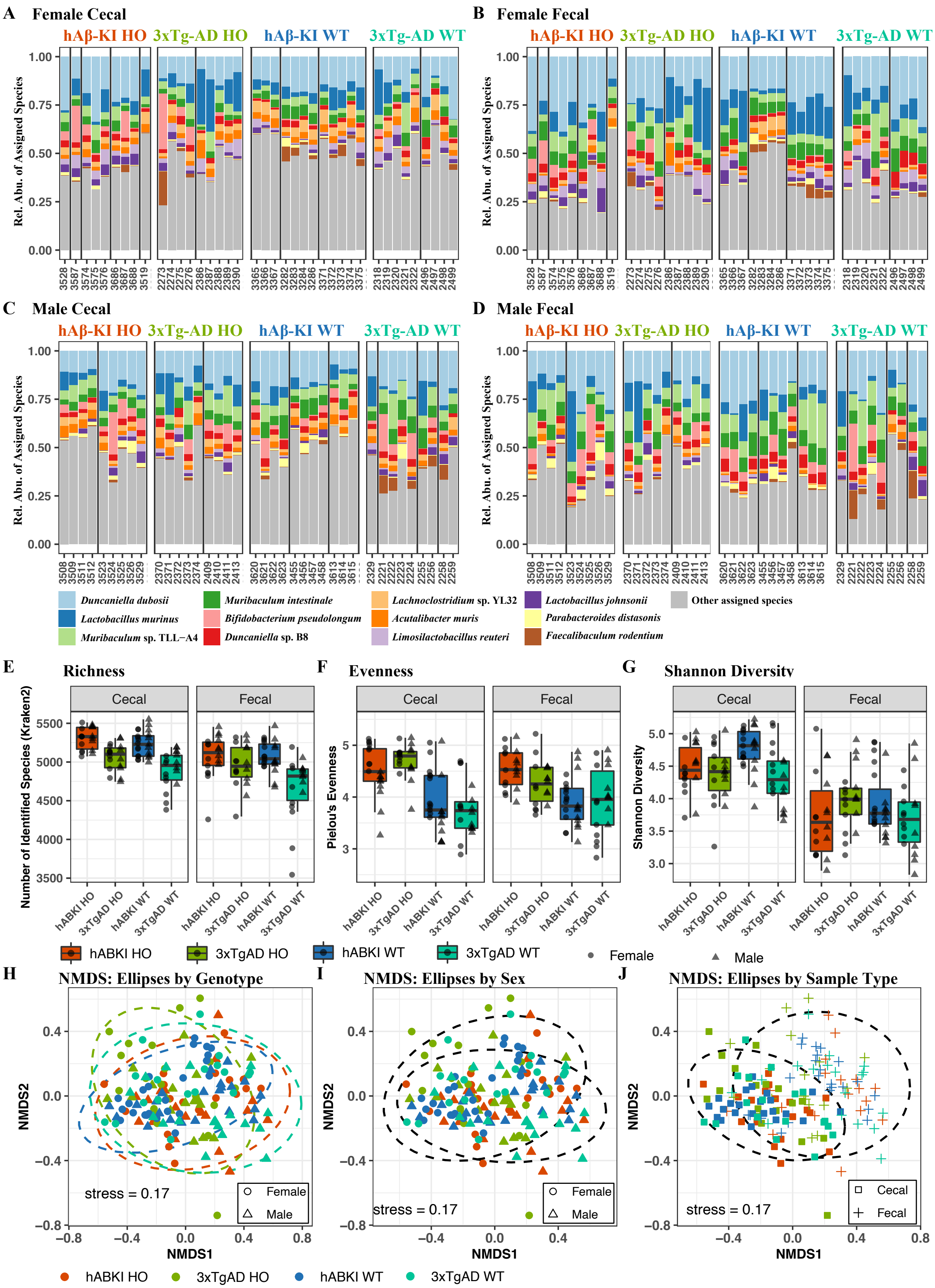

A Female Cecal
B Female Fecal
C Male Cecal
D Male Fecal
hAβ-KI HO
3xTg-AD HO
hAβ-KI WT
3xTg-AD WT
Rel. Abu. of Assigned Species
Duncaniella dubosii
Lactobacillus murinus
Muribaculum sp. TLL−A4
Muribaculum intestinale
Bifidobacterium pseudolongum
Duncaniella sp. B8
Lachnoclostridium sp. YL32
Acutalibacter muris
Limosilactobacillus reuteri
Lactobacillus johnsonii
Parabacteroides distasonis
Faecalibaculum rodentium
Other assigned species
E Richness
F Evenness
G Shannon Diversity
Cecal
Fecal
Number of Identified Species (Kraken2)
Pielou's Evenness
Shannon Diversity
hABKI HO
3xTgAD HO
hABKI WT
3xTgAD WT
Female
Male
H NMDS: Ellipses by Genotype
I NMDS: Ellipses by Sex
J NMDS: Ellipses by Sample Type
NMDS1
NMDS2
stress = 0.17
Cecal
Fecal

Figure S7. Species-level taxonomy (**A-D**), alpha diversity (**E-G**), and beta diversity (**H-J**) of 18 month hAβ-KI and 3xTg-AD microbiomes segregated by sex and sample type (cecal and fecal). The x-axis in **A-D** is labeled with the mouse ID and each black box surrounds co-housed mice. Beta diversity is shown by the NMDS of the Bray-Curtis dissimilarity matrix. hAβ-KI WT mice are the genetic background for hAβ-KI (B6J) and 3xTg-AD mice are the genetic background for 3xTg-AD (B6129).

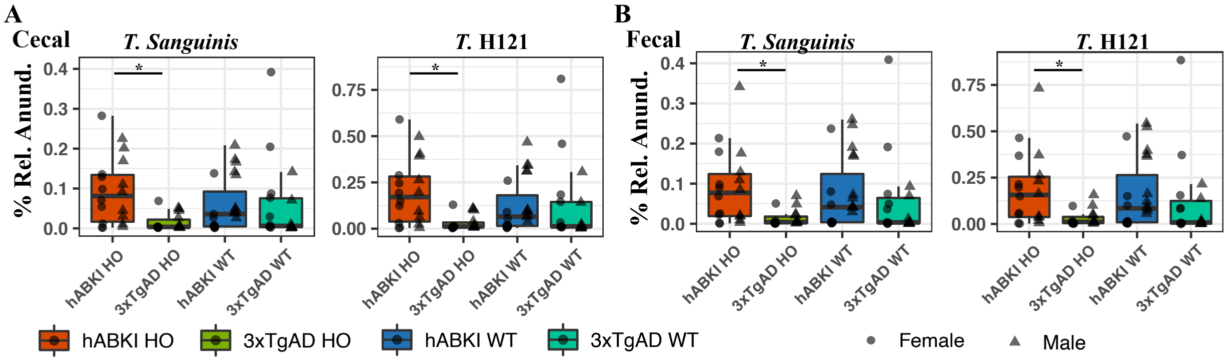

Figure S8. Analysis of *Turicibacter* spp. relative abundance. p-values were Benjamini & Hochberg FDR corrected for 8 repeated measures. *padj < 0.05; padj > 0.05 for all other group means.
